# Accelerating the performance of district health systems towards achieving UHC via twinning partnerships

**DOI:** 10.1186/s12913-020-05741-1

**Published:** 2020-09-21

**Authors:** Mesele Damte Argaw, Binyam Fekadu Desta, Mengistu Asnake Kibret, Melkamu Getu Abebe, Wubishet Kebede Heyi, Elias Mamo, Tesfaye Gebru, Chala Gelan, Bekele Belayhun Tefera, Temesgen Ayehu Bele

**Affiliations:** 1 USAID Transform: Primary Health Care project, JSI Training & Research Institute, Inc. in Ethiopia, P.O. Box 1392 code, 1110 Addis Ababa, Ethiopia; 2 USAID Transform: Primary Health Care project, Pathfinder International, Addis Ababa, Ethiopia; 3 Federal Ministry of Health, Health Extension and Primary Health Services Directorate, Addis Ababa, Ethiopia

**Keywords:** Twinning partnership, Universal health coverage, Transformation, Ethiopia

## Abstract

**Background:**

A twinning partnership is a formal and substantive collaboration between two districts to improve their performance in providing primary healthcare services. The ‘win-win’ twinning partnership pairs are categorized under relatively high and low-performing districts. The purpose of this formative evaluation is to use the empirically derived systems model as an analytical framework to systematically document the inputs, throughputs and outputs of the twinning partnership strategy.

**Methods:**

This explanatory sequential mixed method study design was conducted from October 2018 to September 2019, in Amhara, Oromia, Southern, Nations, Nationalities and Peoples’ (SNNP) and Tigray regions. The quantitative research approach used an observational design which consists of three measurements: at baseline (October 2018), midterm (March 2019) and end-line (September 2019), and the qualitative approach employed a case study. Qualitative data was collected using interviewer-guided semi-structured interview tools. The data were transcribed verbatim, translated from Amharic and Afan Oromo into English and analyzed through a theoretical framework named the Bergen Model of Collaborative Functioning (BMCF). Quantitative data were extracted from routine health management information system. The results are presented as averages, percentages and graphs. To claim statistical significance, non-parametric tests: Friedman test at (*p* < 0.05) and Wilcoxon signed ranks test (*p* < 0.017) were analyzed.

**Results:**

The District Health System Performance (DHSP) was determined using data collected from eight districts. At baseline, the mean DHSP score was 50.97, at midterm, it was 60.3 and at end-line, it was 72.07. There was a strong degree and statistically significant relationship between baseline, midterm and end-line DHSP scores (r > 0.978**), using the Friedman test χ^2^(2) = 16.000, *p* = 0.001. Post hoc analysis using Wilcoxon signed-rank test was conducted with a Bonferroni correction and the results elicit higher DHSP values from baseline to midterm and from midterm to end-line with significance level set at *p* < 0.017. The qualitative results of the case study revealed that scanning the mission of the twinning partnership and focusing on a shared vision coupled with mobilizing internal and external resources were the fundamental input elements for successful twinning partnerships at the district level. In addition, the context of pursuing Universal Health Coverage (UHC) through achieving transformed districts can be enhanced through deploying skilled and knowledgeable leadership, defining clear roles and responsibilities for all stakeholders, forming agreed detailed action plans, and effective communication that leads to additive results and synergy. The twinning partnership implementing districts benefit from the formal relationship and accelerate their performances towards meeting the criteria of transformed districts in Ethiopia.

**Conclusions:**

Twinning partnerships help to accelerate the health system’s performance in achieving the district transformation criteria. Therefore, scaling up the implementation of the twinning partnership strategy is recommended.

## Background

Ethiopia has successfully achieved the maternal and child health targets set in the Millennium Development Goals (MDGs), which have been implemented for the past two decades - from 1995 to 2015. During this period, the government of Ethiopia has put in place a national health policy and several health reforms that have improved the overall performance of the health system. Government commitment, the support of development partners and community-level engagement have contributed to improved and remarkable health outcomes [1, 2]. Some of the gains realized by 2016 include a reduction in maternal mortality ratio (MMR), from 1400 to 351/100000 live births and a decline in the under-five mortality rate by 67, to 68 deaths per 1000 births [3]. The current Health Sector Transformation Plan (HSTP) strategizes to maintain the country’s success towards realizing Universal Health Coverage (UHC) by implementing four transformation agendas that consist of: [1] quality and equity of health services, [2] Caring, Respectful and Compassionate (CRC) health workforce, [3] information revolution, and [4] woreda transformation [3]. This requires closing the gap between high and low-performing district (*woreda*) health offices and adopting and implementing innovative service delivery and management solutions [3] as one of the major obstacles to transformation is the wide variance of know-how, skills, competencies and performances within woreda health systems and/or among woredas within zone administrations and regions.

## The case

The USAID Transform: Primary Health Care project is targeting 400 districts; 95 (23.7%) of which are in Amhara; 162 (40.5%) are in Oromia; 120 (30.0%) are in SNNP; and 23 (5.7%) are in Tigray regions. Within these project targeted regions there are: 114 primary hospitals; 1837 health centers; and 9538 health posts [4]. The Ministry of Health has defined a set of criteria that include district *(woreda)* management standards (10.0%), model villages (30.0%) high performing primary health care units (30%), and financial risk protection through high membership and renewal coverage of the community-based health insurance scheme (30%). Using the overall collated scores, districts that achieved greater or equal to 80% are considered high performers; districts that achieved between 60 to 79.9% are medium performers and districts that achieved less than 60% are low performer districts. By the end of September 2018, out of 1081 districts, no districts had achieved 80% or more.

In 2017, the Ethiopian Federal Ministry of Health (FMOH), and Regional Health Bureaus (RHBs) in collaboration with the project piloted the ‘twinning partnership strategy’ to accelerate district health system performances as a result of synergy, that is, district transformation as an outcome through working with others, which leads towards UHC [5–11].

According to Cadée et al.*,* 2016, twinning is a cross-cultural, reciprocal process in which two groups of people work together to achieve joint goals [12]. Similarly, twinning is defined as a formal and substantive collaboration between two organizations [6, 10]. Formal means that there is a verbal or written agreement between the two organizations. Substantive means that the interaction is significant, and that it lasts for a specific period i.e., it is not a one-time interaction. Collaboration means that the two organizations work together on a specific project or to exchange information or skills [7].

**Fig. 1:**
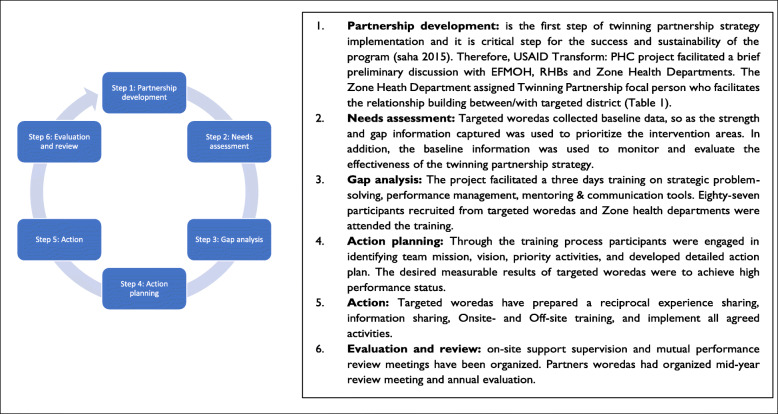
Partnership implementation cycle (6). The figure depicts six steps of partnership implementation cycle and detailed descriptions of activities followed at each stage

The foundation of established twinning partnerships follows a ‘win-win’ mutual relationship and jointly understood principles. In addition, the twinning partnership is based on proven willingness and commitment between woredas within different performance tiers to collaborate for at least one full year [6]. After conducting preliminary discussions with stakeholders, a memorandum of understanding between both districts and zonal health departments was signed. A comprehensive need assessment was conducted, and gaps were identified. Then, 187 delegates of partnering districts participated in a three-day basic twinning partnership strategy training and developed a twinning partnership project for one year (Fig. 1). During the activity implementation phases, partnering districts are expected to mobilize resources, share information, organize learning experiences, exchange experts, conduct on-site and off-site trainings, monitor implementation of action plans and organize common knowledge sharing collaborative workshops.

Multiple studies have documented twinning partnerships between research institutes in low income countries and middle/high income countries located in Europe, North America and South Africa [11–16]. Similarly, there is ample documented evidence on prompting health behaviors through north-south partnerships [17–20]. In addition, to narrow the performance differences, the project developed and innovative way to strengthen capacities and skills of the healthcare workforce through establishing in-country twinning partnerships between districts in Ethiopia [6, 21].

**Fig. 2:**
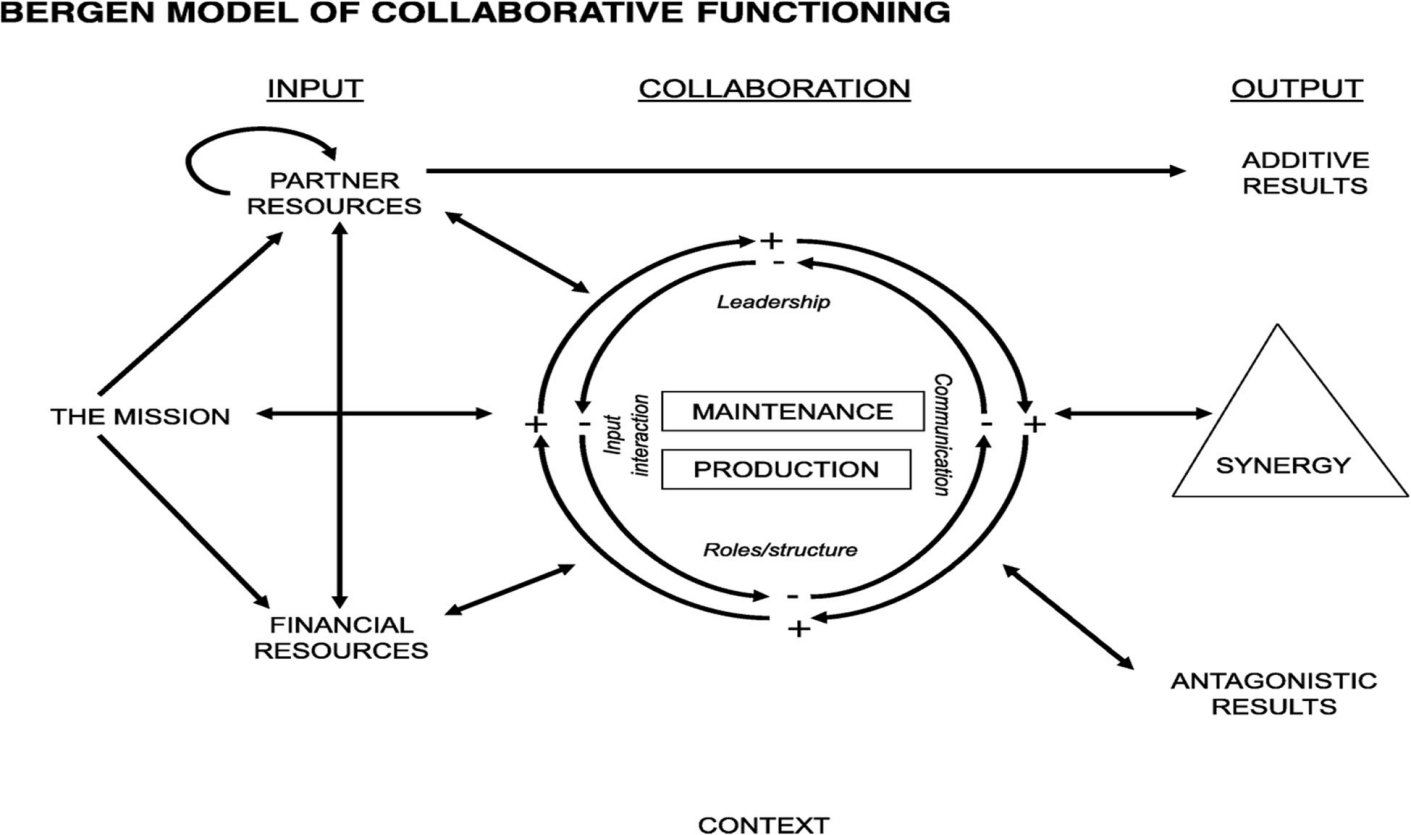
The Bergen Model of Collaborative Functioning [19, 20]. BMCF conceptual map adopted to evaluate the twinning partnership strategy implementation in Ethiopia. The framework depicts the relations of inputs, throughputs and outputs of collaborative functioning

The main purpose of this formative evaluation is to systematically document the process of the twinning partnership strategy implementation and evaluate the effects of implementing the twinning partnership strategy on accelerating district transformation and to compare the results of the intervention over time using an empirically derived systems model as an analytical framework to systematically document the input, throughput and output parameters of the BMCF theoretical framework (Fig. 2), in Ethiopia, where the twinning partnership strategy is being implemented between districts targeting UHC by achieving district transformation. Therefore, this research was conducted to answer research questions: how does the twinning partnership strategy accelerate district health system performances towards achieving UHC in Ethiopia? And, what factors influence the outputs of the twinning partnership strategy in USAID Transform: Primary Health Care project targeted districts in Ethiopia?

## Methods

### Study design, setting and population

An explanatory sequential mixed method study design was conducted in eight districts of Amhara, Tigray, Oromia and SNNP regions. The quantitative research approach consists of three measurements at baseline, midterm and end-line, which allowed the investigators to measure the effects of implementing twinning partnership strategy on performances of the district health system. The qualitative research approach uses a case study design [22]. The investigator chose a case study design to explore the implementation of the twinning partnership strategy and collect detailed data from various sources. Similarly, Yin (2014) confers that in a case study, evidences may come from multiple sources, namely: documents, interviews, direct observation, and physical artifacts [22]. The total population of the study area were about 970, 625 people with 3 primary hospitals, 37 health centers and 164 health posts. The study was conducted from October 2018 to September 2019 and focused only on eight districts that had implemented the twinning partnership strategy for one full year.

### Quantitative: population, sampling and sample size

In September 2018, the eight districts were categorized into three performance levels. The performance categories were five low performing and three medium performing districts. The sampling method was purposive as the districts chosen had to have had a history of implementing the twinning partnership strategy aiming to achieve UHC through achieving district transformation in Ethiopia. Districts either not familiar with twinning partnership strategy or those that were in the process of implementing their action plan for less than six months were excluded. The districts sampled and enrolled in this study were: Machakel (medium performer) and Bibugn (low performer) in the Amhara region; Damboya (medium performer) and Hadero Tunto Bibugn (low performer) in the SNNP region; Chelia (low performer) and Elu-Gelan (low performer) in the Oromia region; and Raya Azebo (medium performer), and Ofla Bibugn (low performer) in the Tigray region [21].

### Quantitative data collection and management

The quantitative data which were extracted using nationally developed self-assessment and validation tools were aligned with Ethiopia’s Ministry of Health measurements of performance status of district health systems. The questionnaires were developed in English. The data were extracted from routine health management information system reports and reports of external validation teams. There are four transformation measurement parameters that determine the categories of districts, namely: woreda (district) management standards, high performing primary health care units (PHCUs), model villages (kebeles) and community-based health insurance. District management standards are measured against 27 standards and 87 validation indicators (additional file 1). Model villages are measured against four standards, namely: model household coverage, improved latrine coverage, facility delivery coverage and percentage of model schools and has 28 composite validation indicators (additional file 2). Community-based health insurance is measured by the coverage of households with an active membership captured as new enrollment and annual renewal rates of households within a defined catchment population (additional file 3). High Performing PHCUs are measured using 28 standards of the model village measurements (30.0%), key performance indicators with 18 parameters (35.0%), (additional file 4) and the Ethiopian health center reform implementation guidelines which contain 81 standards with 209 composite validation indicators (35.0%), (additional file 5). In order to translate the scores to percentage of standard met, a score of one is given to each standard when all validation criteria are met, otherwise a score of zero was given. The overall summary of district health system performance is reported as a percentage from 0.0–100.0 on three occasions.

The baseline, midterm and end-line data were collected in October 2018, March 2019 and September 2019 from all eight district health offices, respectively. The data were collected by eight trained health professionals with a master’s level of education. Data were entered and cleaned using the Microsoft Excel computer program. The completeness and consistency of the data were checked and exported into the Statistical Packages for Social Science version 20.0 for Windows (SPSS, Chicago, IL, USA).

### District performance determination

Quantitative data analysis methods were used which included descriptive statistic frequencies, mean, median, interquartile ranges, standard deviation, and graphs. To determine the presence of a linear relationship between baseline, mid-term and end-line scores, the Pearson product-moment correlation was tested. Then, the following assumptions of Friedman’s [23] non-parametric test was checked. These include: [1] a single group measured on three or more different occasions; [2] a group that is a random sample from the population; [3] a continuous level of dependent variables; and [4] sample do not need to be normally distributed. Post hoc analysis using the Wilcoxon signed-rank test was conducted with a Bonferroni correction applied and a statistical test result with a *p*-value of less than < 0.017 indicated the presence of a significant difference between candidate variables at baseline, midterm and end-line measurements [23].

### Qualitative: population and sampling

The study population for the qualitative approach was 187 health workers and managers who were trained and had implemented the twinning partnership strategy in their respective health facilities. The study used purposive sampling methods. Firstly, four regional states and eight districts were selected based on their experience of implementing the twinning partnership strategy. Secondly, 39 In-depth Key Informants (IKI), of which six were female were enrolled until data saturation was reached, as a result of redundancy of information.

### Qualitative data collection and management

The in-depth- interview guide was adopted from previous published researches [17, 24]. The interview guides were developed in English and translated into local languages of Amharic and Afan Oromo (additional file 6). The tools were then piloted in two selected districts located in Amhara and Oromia regions. The pilot districts were implementing the twinning partnerships strategy for six months. Before developing the final data collection tools, the necessary amendments were made based on pilot test results. The pilot testing data were not included in the final study. The data were collected using in-depth individual interviews with healthcare professionals of primary health care entities who were actively engaged in the implementation of the twinning partnership strategy. The main questions were: ‘*Would you tell me the process you followed in implementing the twinning partnership strategy at your organization?’, ‘Have you observed changes in the health system as result of the implementation of the twinning partnership?’, ‘What are the changes?’, and, ‘What do you recommend in accelerating the performance of the district health system using the twinning partnership approach?’*. The qualitative data saturation was reached with no new ideas and themes after interviewing 39 health workers.

### Qualitative data analysis

The qualitative data analysis was conducted concurrently with data collection. During the data collection, all interviews were recorded using digital voice recorders (ICD-BX140), thirty-nine transcribed verbatim - 29 in Amharic and 10 in Afan Oromo, were translated into English. To interpret the data, two investigators read and re-read the transcripts several times for better understanding. Emerging ideas were written and codes were created with grouping and regrouping of codes to topics with corresponding similarities [25]. In addition, the Bergen Model of Collaborative Functioning (BMCF) theoretical framework was used as a basis for interpreting the findings [19, 20]. The data and BMCF was given to a public health expert to analyze the data independently. A consensus meeting between the principal investigator and a public health specialist was held, where the codes and themes were compared and an agreement was reached on the emerging ideas.

### Measures to ensure trustworthiness

Credibility, dependability, transferability, and confirmability were maintained to ensure the true value of the study’s findings [26, 27]. The strategies used to ensure credibility of the study were prolonged engagement in the field which provide the data collectors an adequate time to build trust, conduct persistent observations, clarify misinformation and ensure saturation of data. Individual transcripts were shared with each in-depth interviewee and spot checks were conducted to capture additional opinions, experiences and remove misinformation or distortions. Using different data sources, and through extracting data on three occasions in a year, the data triangulation was conducted. In addition, the qualitative data codes, themes and interpretations were triangulated between two researchers. The dependability of this study was ensured through implementing a strategy called the audit trial. The research steps were clearly described from protocol development to reporting the findings. All transcripts, audio records, hand written reports and the extracted data were shared among data collectors and supervisors. All data were also shared with public health specialists to check the dependability of the process. The transferability of this research was ensured through implementing thick description as a strategy. The investigators described the twinning partnership in the context of achieving UHC through increasing transformed districts in Ethiopia. Furthermore, thick descriptions were provided about the informants, experiences and process observed during the study. The confirmability of this study was ensured through audit trial. In addition, the findings of this study was controlled with literatures.

## Ethical considerations

The study protocol was reviewed at the JSI Research & Training Institute, Inc. Institutional Review Board (IRB). The IRB has determined that this activity is exempted from human subjects’ oversight (IRB #19-31E). Support letters were sought and obtained from the Amhara, SNNP, Oromia and Tigray regional state health bureaus. Permission to conduct the study was sought from the selected health facilities and informed written consent was obtained from all study participants. All study subjects whose age is greater or equal to 18 years were informed that they have the right to discontinue or refuse to participate in the study at any time. The investigator has maintained the anonymity, privacy and confidentiality of the participants throughout the research process.

## Results

The results of this study are presented as follow as characteristics of selected districts, performance of districts and quantitative findings.

### Quantitative study findings

Characteristics of selected districts: quantitative data were collected from eight districts in four regional states of Ethiopia. On average, there are about 121,328 inhabitants in each district. Health services are offered to the community through three primary hospitals, 37 health centers, 164 health posts, and 175 primary schools (Table 1).

**Table 1:** Characteristics of selected districts (woredas) in the study area, September; 2019.

Region	District	Distance form regional capital in Km	Population	Number of Households	Number of Primary Hospitals	Number of Health Centers	Number of Health Posts	Number of schools
Oromia	Illu Gellan	210	86,006	19,112	0	3	18	19
	Chelia	180	108,000	24,000	1	4	20	22
SNNP	Damboya	110	108,359	24,080	1	4	19	19
	Hadro Tunto	140	107,644	23,921	0	3	20	22
Amhara	Machakel	230	141,167	31,370	0	6	27	30
	Bibugn	320	96,137	21,364	1	4	18	19
Tigray	Raya Azebo	250	170,103	37,801	0	7	17	18
	Ofla	270	153,209	34,046	0	6	25	26
Overall	Average	214	121,328	26,962	0.38	4.6	21	22
	Total	1710	970,625	215,694	3	37	164	175

### Performance of district health systems

**Fig. 3:**
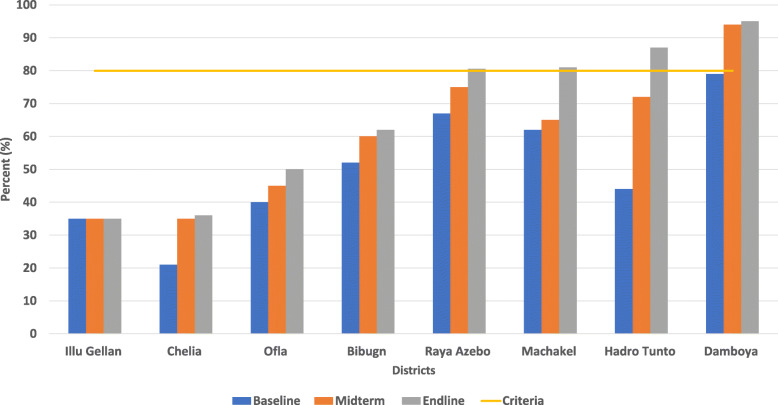
Baseline, midterm and end-line measurements against district transformation criteria among partner districts, October 2018 – September 2019. The bins show the trends of improvement from baseline, to midterm and end-line measurements

All twinning partnership targeted districts were rated on three occasions: baseline, midterm and end-line stages. Figure 3 below depicts the five and three districts that were categorized as low and medium performers at the baseline measurement stage, respectively. These scores were improved at midterm as one, four and three district categories as high, medium and low performing districts, respectively. The end-line results revealed that out of eight districts, four fulfilled the transformation criteria, a district was categorized as a medium performer and the remaining three districts - despite improving their scores, fell in the low performing district category.

Table 2 below shows that the correlation (r) results of district health system performances. There was a strong degree and statistically significant relationship between baseline and midterm (r > 0.978**), baseline and end-line (r > 0.936**), and midterm and end-line (r > 0.987**) scores.

**Table 2:** Correlation summary analysis of each district’s health system performance, October 2018 – September; 2019.

	DHSP	DHSP	DHSP
	Baseline	Mid-term	End-line
District Health System Performance (DHSP) - Baseline			
Pearson Correlation	1		
Sig. (2-tailed)			
N	8		
District Health System Performance (DHSP) - Mid-term			
Pearson Correlation	.978^**^	1	
Sig. (2-tailed)	0.001		
N	8	8	
District Health System Performance (DHSP) - End-line			
Pearson Correlation	.936^**^	.987^**^	1
Sig. (2-tailed)	0.001	0.001	
N	8	8	8

**. Correlation is significant at the 0.01 level (2-tailed)

•Perfect: If the value is near ±1 then it said to be a perfect correlation; as one variable increases, the other variable tends to also increase (if positive) or decrease (if negative).

•High degree: If the coefficient value lies between ±0.50 and ± 1 then it is said to be a strong correlation.

•Moderate degree: If the value lies between ±0.30 and ± 0.49 then it is said to be a medium correlation.

•Low degree: When the value lies below + 0.29 then it is said to be a small correlation.

•No correlation: When the value is zero.

### Difference in woreda management standard scores over time

At baseline, the mean score in woreda management standards (WMS) was 6.42, at midterm, it was 7.52, and at ‘end-line’, it was 8.15. There was a statistically significant difference in WMS scores at baseline, midterm and end-line measurements, χ^2^(2) = 14.250, *p* = 0.001. Post hoc analysis using the Wilcoxon signed-rank test was conducted with a Bonferroni correction applied, resulting in a significance level set at *p* < 0.017. Median (IQR) WMS at baseline, midterm and end-line were 6.45 (5.35 to 7.42), 7.65 (6.25 to 8.85), and 7.95 (6.95 to 9.30), respectively. There were statistically significant positive differences between the WMS2 – WMS 1 (*Z* = − 2.524, *p* = 0.012), WMS3 – WMS2 (*Z* = − 1.963, *p* = 0.050), and WMS3 – WMS1 (*Z* = − 2.521, *p* = 0.012).

### Difference in model village scores over time

At baseline, the mean score of model villages (MV) was 11.58, at midterm, it was 14.88, and at ‘end-line’, it was 20.85. There was a statistically significant difference in MV coverage at baseline, midterm and end-line measurements, χ^2^(2) = 16.000, *p* = 0.001. Post hoc analysis using the Wilcoxon signed-rank test was conducted with a Bonferroni correction applied, resulting in a significance level set at *p*- < 0.017. Median (IQR) MV coverage at baseline, midterm and end-line were 10.65 (7.12 to 18.65), 14.56 (9.52 to 21.37), and 22.05 (14.70 to 26.32), respectively. There were statistically significant positive differences between the MV2 – MV1 (*Z* = − 2.533, *p* = 0.011), MV3 – MV2 (*Z* = − 2.527, *p* = 0.012) and MV3 – MV1 (*Z* = − 2.524, *p* = 0.012).

### Difference in high performing PHCU scores over time

At baseline, the mean score of high performing PHCUs (HP PHCU) was 17.96, at midterm, it was 20.43, and at ‘end-line’, it was 23.32. There was a statistically significant difference in HP PHCU scores at baseline, midterm and end-line measurements, χ^2^(2) = 11.677, *p* = 0.003. Post hoc analysis using the Wilcoxon signed-rank test was conducted with a Bonferroni correction applied, resulting in a significance level set at *p* < 0.017. Median (IQR) HP PHCU at baseline, midterm and end-line were 17.10 (14.02 to 22.57), 20.55 (15.97 to 24.22), and 22.80 (19.35 to 28.12), respectively. There were statistically significant positive differences between the HP PHCU2 – HP PHCU1 (*Z* = − 1.960, *p* = 0.050), HP PHCU3 – HP PHCU2 (*Z* = − 2.536, *p* = 0.011), and HP PHCU3 – HP PHCU1 (*Z* = − 2.366, *p* = 0.018).

### Difference in community-based health insurance scores over time

At baseline, the mean score of community-based health Insurance (CBHI) was 15.0, at midterm it was 17.51, and at ‘end-line’, it was 19.74. There was a statistically significant difference in CBHI coverage at baseline, midterm and end-line measurements, χ^2^(2) = 13.556, *p* = 0.001. Post hoc analysis using the Wilcoxon signed-rank test was conducted with a Bonferroni correction applied, resulting in a significance level set at *p* < 0.017. Median (IQR) CBHI coverage at baseline, midterm and end-line were 14.40 (10.87 to 19.72), 18.75 (11.25 to 22.27), and 21.39 (11.85 to 25.65), respectively. There were statistically significant positive differences between the CBHI2 – CBHI1 (*Z* = − 2.207, *p* = 0.027), CBHI3 – CBHI2 (*Z* = − 2.371, *p* = 0.018), and CBHI3 – CBHI1 (*Z* = − 2.371, *p* = 0.018).

### Difference in overall district health system performance scores overtime

At ‘baseline’ the mean score of district health system performance (DHSP) was 50.97, at ‘midterm’ it was 60.3, and at ‘end-line’, it was 72.07. There was a statistically significant difference in district health system performances at baseline, midterm and end-line measurements, χ^2^(2) = 16.000, *p* = 0.001. Post hoc analysis using the Wilcoxon signed-rank test was conducted with a Bonferroni correction applied, resulting in a significance level set at *p* < 0.017. Median (IQR) district health system performance measurements at baseline, midterm and end-line were 47.55 (36.10 to 67.32), 60.50 (42.3 to 74.18), and 72.99 (52.28 to 89.25), respectively. There were statistically significant positive differences between the DHSP2 – DHSP1 (*Z* = − 2.521, *p* = 0.012), DHSP3 – DHSP2 (*Z* = − 2.521, *p* = 0.012), and DHSP3 – DHSP1 (*Z* = − 2.524, *p* = 0.012). Therefore, it can be concluded that the performances of district health systems were significantly raised over time.

### Qualitative study findings

Data collected from health workers with diverse professional backgrounds: health officers (14; 35.9%), nurses (9; 23.1%) and midwives (2; 5.1%) were used for the study. The majority 33 (84.6%), were male. The mean age with standard deviation was 28.5 ± 5.0 years. On average, 7.5 years of service were tenured by the KIIs (Table 3).

**Table 3:** Socio-demographic Characteristics of Key Informants, (*N* = 39), September 2019

Characteristics	Number (%)
Region	
Tigray	7 (17.9)
Amhara	8 (20.5)
Oromia	10 (25.6)
SNNP	14 (35.9)
Gender	
Male	33 (84.6)
Female	6 (15.4)
Profession	
Health Officer	14 (35.9)
BSc. Nurse	9 (23.1)
Laboratory Technologist	5 (12.8)
Master’s in Public Health	3 (7.7)
BA in Economics	2 (5.1)
Midwife	2 (5.1)
Health Extension Worker	2 (5.1)
Pharmacy Technician	1 (2.6)
Health Information Technologist	1 (2.6)
Environmental Health Officer	1 (2.6)
Age category	
< 25 years	8 (20.5)
26–35 years	29 (74.4)
36–45 years	1 (2.6)
46+ years	1 (2.6)
Mean = 28.5 Years; SD = 5.0 Years; Median 27 years and range 27 Years.	
Work experience	
< 3 years	7 (17.9)
4+ years	32 (82.1)

Mean = 7.5 Years; SD = 5.0 Years; Median 6 years and range 27 Years

## Inputs

Based on BMCF, three major categories were discussed under inputs. The categories discussed below are mission, partner resources and financial resources.

### Mission

In this study, ‘mission’ means the main reason stated for the existence of the twinning partnership. A common understanding of the mission and the health system’s strategic priorities enhance access to quality primary healthcare services in an equitable manner. Such systematic interventions help the Ethiopian health sector create a resilient district health system that is responsive to the needs and demands of all individuals in all places. These collective and widespread comprehensions help members of the twinning facilities to develop a shared vision that inspires partner districts and their staff, share resources, develop a culture of serving communities outside their district boundaries, and grow and become stronger together while maintaining a sense of competitiveness among members. This indicates that the implementation of health sector reforms enhance the governance, capacity, quality, and equity of access to primary health care services. The following verbatims describe the opinions of health workers on their understanding of the mission and shared vision of the twinning partnership strategy.

*“Although we, [members of twinning partnership] live in neighboring woredas and are familiar with each other as health workers, we had never talked with each other about enhancing our health services. If fact, we were reluctant to share information as we wanted to stand out and be better performers than other health centers located in the same [name] zone. The twinning partnership approach helped us to open our eyes and broadened our horizons. More specifically, we understood that through working together, we became stronger. Hence, the partnership helped us get closer to our ultimate goal of serving the community.”* (004, Health Center Director, Health Officer, Oromia region)

Some of the twinning partnership districts developed a vision or mission statement that supports the achievement of the Ethiopian health system long term goal of achieving UHC by increasing the coverage of transformed districts*: “…to create model primary health care units in both [name] and [name] districts.”* (033, District Health Office Head, Master of Public Health, Amhara region). Another staff member commented that their vision was, *“… to be a transformed district.”* (001, Health Center Director, Health Officer, Oromia region).

A health center staff also expressed his observations on the benefits of empowering health workers through a shared vision: *“Staff at our partner district were familiar with the health sector reforms. Therefore, we created a platform for our districts to organize a number of seminars for staff that assisted us in creating a shared vision.” (*010, Vice head of Health Center, Health Extension worker, Oromia region).

Another health professional commented on the impact of having shared missions and visions *saying; “Previously, other sector heads considered health as a well-financed sector through development partners. However, after visiting the health centers in person, they recognized that health is an expensive service for many beneficiaries who expressed several grievances. To fulfil the minimum standards, ownership and local financing can help the health system to achieve its major goal of preventing maternal and child deaths.”* (012, Reform Core Process Owner, BA in Economics, Tigray region).

### Partner resources

Narrowing the performance gaps of districts in a short time demands the utilization of internal and external resources. The twinning partnership strategy was facilitated by three to four committed staff members assigned from the district health offices and health centers. Zone Health Department (ZHD) assigned focal persons who liaise between districts. In addition, focal persons were assigned to each department such as the laboratory, pharmacy, health center - health post linkage, health information system, quality improvement and infection prevention in each partnering district. The majority of the respondents believed that having a dedicated partnering facility helped them adopt innovative tools and achieve better results. One of the respondents commented, *“If it wasn’t for the technical support on facilitating the preliminary discussions, partnership development, facilitating basic twining partnership trainings, developing problem solving skills, partner districts would have continued doing things as usual.”* (021, Health Center Director, Environmental Health Officer, SNNP region).

### Financial resources

This broad category addresses the effort of twinned districts in mobilizing monetary resources. Experience sharing between medium and low performing districts helps executive and decision-making bodies understand the concept of performance management by institutionalizing minimum standards. A district health office staff member explains, *“The experience sharing and learning tour helped the executive team understand the idea of achieving universal health coverage through district transformation and enabled them to compare our district’s achievements to that of our neighboring twin woreda, [name] district. They, [executive body] committed themselves to replicate what they saw in [name] Health Center and allocated $66,000.00 (sixty-six thousand USD) for availing essential drugs and the renovation of health facilities.”* (037, District Health Office Head, BSc in Nursing, Amhara region).

Another district health office staff had this to say about the financial support they received from a development partner: *“Without the financial support we received from the project, we might not be familiar with the concept of the twinning partnership which led us to develop projects and engage in the implementation of activities.”* (018, District Health Office Head, master’s in public health, SNNP region).

Therefore, scanning health sector priorities and the establishment of missions led the participants to develop a shared vision of serving communities that motivated them to mobilize the necessary human, financial and other resources. These factors were the key elements of the inputs in the implementation of the twinning partnership strategy in Ethiopia.

## Collaboration

Five major categories were presented under the theme of collaboration. The emerged categories discussed below are input interaction, leadership, formal roles and procedures, communication, and maintenance task.

### Input interaction

The results of scanning missions, developing shared visions, mobilizing financial, human and other resources were steps of the collaboration that established inputs for the interactions. The majority of the respondents believed that the shared missions, shared visions, interactions with partners and allocation of financial resources had a positive effect on accelerating the performances of primary health care units and the district health system.

One of the district health office heads had this to say: “…s*taff mobilization and deployment, allocation of budget for fuel, covering staff accommodation costs and sharing of resources in the twinning partnership assisted us to motivate staff and led to the achievement of our shared vision.”* (011, District Health Office Head, BSc nurse, Tigray region).

Similarly, a head of a health center expressed*, “Our health center ran out of lab supplies for syphilis screening tests, and the antenatal services were not complete for pregnant mothers. Similarly, our partner district reported an unusual increase in the number of malaria cases observed in health facilities, while they lack antimalaria drugs. Both of us benefited from the established relationship as we were able to share human resources and essential drugs and supplies.”* (034, Health Center Director, BSc Nurse, Amhara region).

#### Leadership

The capacity of the leadership to scan their environment, focus on impactful interventions, exhibit motivating and inspiring behavior, and mobilize and align resources were pointed out as having a positive influence on the achievement of twinning partnership projects. A health worker affirms, “*Assessing the needs and demands of the district health system’s environment, mobilizing and aligning resources coupled with leaders’ recognition and acknowledgement of staff cemented commitment for the twinning partnership.”* (017, Health Center Director, Health Officer, Tigray region).

The majority of the respondents believed that the commitment of the assigned focal persons in planning, organizing, facilitating, implementing and monitoring the activities had a positive effect on the established partnerships. Almost all staff who were engaged in the twinning partnership were acknowledged by the top managers as having facilitated vehicles, per-diem and other resources effectively which led to the successful implementation of the developed twinning projects.

#### Formal roles and procedures

Each level of the healthcare system maintains a set of roles and responsibilities endorsed by partners ensuring the implementation of the twinning strategy at all levels of the health system. The majority of the respondents highlighted that the established clear structures, roles and responsibilities of all stakeholders helped them to achieve better results. One of the district health office managers had this to say: *“The launching workshop showed us the structures, roles and responsibilities of the twinning partnership strategy. We also defined and shared this at the district health office and health center level which was instrumental for effectively carrying out our twinning partnership planned activities.”* (*002*, District Health Office Head, BSc Nurse, Oromia region). Committed and willing districts and zone health departments signed a memorandum of understanding to work together for one full year*.*

#### Communication

During the implementation of the twinning partnership, project members communicated with each other through various channels including telephone conversations, written communication, creating groups through mobile phone applications, and through face to face communication. A health worker had this to say about improving performance of partners using telephone communication*: “We usually use telephone communication to arrange meetings, experience sharing events, and invite experts. This helped us to maintain our friendly relationship.”* (012, Health Reform Core Process Owner, BA in Economics, Tigray region).

Another health worker described the importance of face to face communication supplemented by written letters on sharing of drugs and supplies: *“If we were requesting drugs and supplies we used formal letters along with face to face communication”* (005, Head of Health Center - Pharmacy Department, Pharmacy Technician, Oromia region). The health manager explains how a mobile phone application was used to create groups for information sharing on the performance of the district’s health system. *“We have a Telegram [application] group where we update each other on our day to day performances.”* (028, Maternal and Child Health expert, Health Officer, SNNP region).

Another health center staff had to say: *“Before we engaged in the twinning partnership, our communication was limited within district health teams as we had no means of sharing experience and supporting each other. We used to mainly meet during review meetings where we share and learn about experiences and successes. We also did not have the means to organize learning tours to other primary health care facilities. Now, our health center [name] formally communicates with the twinned health center located in [name] district.”* (001, Health Center Director, Health Officer, Oromia region).

#### Maintenance task

The basic twinning partnership training focuses on four main chapters, namely: health sector priorities, strategic problem solving, performance management and communication. During the implementation of the twinning partnership, the importance of clear reporting requirements and of sharing information were well addressed. One of the district health office staff had this to say: “*During the basic twinning training, participants identified the current situation, developed desired measurable results, identified obstacles and prioritized solutions. In addition, a detailed action plan on resource mobilization as well as monitoring and evaluation was prepared. These activities helped us to share basic information and performance status.*” (032, District Health Office Vice Head, BSc Nurse, Amhara region).

Another staff member explained how the achievements were garnered through integration with routine health system activities: “*We organized supportive supervision, learning tours, and facilitated a number of workshops on the Ethiopian primary health care alliance for quality.*” (023, Health Center Staff, Midwife, SNNP region),

A health center staff also expressed his opinion on effective maintenance: *“The staff facilitated onsite and off-site trainings, experience sharing events, common review meetings and expert exchange platforms.”* (038, Health Center Quality Improvement Officer, master’s in public health, Amhara region).

## Outputs

Three major categories were discussed under outputs. The summary of additive results, synergy, and antagonistic results will be presented below.

### Additive results

Additive results implied the implementation of health sector priorities separately, without considering the effects of implementing the twinning partnership strategy. The majority of twinning partnership targeted districts reported their engagement through orientation of health sector reforms, facilitated self-assessments against standards and provided routine health services. A district health office head said, *“…the health office organizes and facilitates orientation of health sector reforms.”* (011, District Health Office Head, BSc nurse, Tigray region).

Another health worker had this to say about the routine activities in their office implemented regularly: *“Every quarter, the performance management team assesses performance against the standards.”* (001, Health Center Director, Health Officer, Oromia region).

### Synergy

The implementation of the twinning partnership strategy helped partner districts achieve results that would not have been achieved through the sole efforts of either individuals or districts.

A health worker described the additive results of the established twinning partnership saying*: “…though we had relatively higher performances than our partner low performer district within the partnership, we learned about experiences of implementing challenging interventions from our twin woreda, [name]. We also adopted the best practices and collected lists of items which are essential for maternal waiting homes and audio-visual job aids.”* (034, Health Center Director, BSc Nurse, Amhara region).

### Antagonistic results

Despite the additive results and synergy observed among partnering districts, there were some observed antagonistic results. During the experience sharing events as well as while conducting integrated supportive supervisions, some staff did not know the reason for the established partnership and the investment appeared to them as a waste of resources.

*“While we were motivated to share our knowledge and skills to our partnering district staff, they perceived us as solely having travelled over 90 kilometers to get financial rewards through our meal and accommodation expense payouts.”* (032, District Health Office Vice Head, BSc Nurse, Amhara region)

Some of the health workers pointed out that lack of transparency in decision making and lack of inclusion of all departments in the established twinning partnership had a negative impact on achievements. Furthermore, they describe the demotivating effects of lack of good governance on collaborative efforts.

*“I was one of the active participants in the development of the one-year twinning project. However, it was not clear to some of us, the process through which leaders and managers hand-picked staff for the experience sharing events….”* (005, Health Center Pharmacy Head, Pharmacy Technician, Oromia region)

## Discussion

Twinning partnership is not a new concept. Many literatures have been documented on North-South and South-South model of twinning partnerships. More specifically, the literatures are dedicated to exploring and determining the effects of such partnerships on the knowledge and skill development among members of partners in different cultural contexts [13–20]. However, this study revealed the importance of establishing an in-country twinning partnership strategy to accelerate the performance of a district health system in a low-income county - Ethiopia.

The twinning partnership strategy implemented in four regional states of Ethiopia clearly demonstrates the win-win collaboration functioning between districts that fall under medium and low performance categories. The main purpose of this formative evaluation report is to demonstrate the effects of the twinning partnership on the performance of district transformation which is the main strategy in achieve UHC in Ethiopia. In addition, the research unveiled the positive and negative factors that enhance or deter the outputs of the twinning partnership strategy [19].

The three point in time average overall DHSP scores revealed increasing trends and with statistically significant differences at *p* < 0.017. The woreda district management standard scores, developed to create a resilient district health system increased from 6.47 to 8.15 out of weighted shares of 10, model village scores increased from 11.58 to 20.85 out of weighted shares of 30, high performing primary health care unit scores increased from 17.96 to 23.32 out of weighted shares of 30, and community based health insurance scores increased from 15.0 to 19.74 out of weighted shares of 30. This result could have been achieved as a result of the synergy created between partner districts. This finding was in line with Argaw et al.*,* 2020 who attest that, the twinning partnerships helps districts to work together and achieve a higher performance category within 18 months [21]. Similarly, Argaw et al., (2019) who confer that working against minimum standards improves the performance of primary health care entities in Ethiopia [5].

Scanning the mission of the twinning partnership and primary health care entities and focusing on the health sector’s top priorities in the context of district transformation was helpful to members of the twinning partnership in developing a shared vision. The shared vision helped leaders and managers to persist in achieving results beyond their districts. It also helped the district health systems to identify stakeholders and define structures, roles and responsibilities. A common understanding of the purpose of the twinning partnership assisted the district health system staff in improving their negotiation skills by convincing decision-making bodies to fulfill minimum human, material and other resource needs [28, 29].

The Ethiopian health sector’s strategic plan strives to achieve UHC by transforming households, villages and primary health care units [3]. The most important element of district transformation is having capable health sector leaders. Visionary leaders who have the knowledge and skills of scanning, focusing, inspiring, aligning and organizing resources were identified as the essential element of implementing collaborative functioning. The training offered to staff of districts engaged in twinning partnership helped them to understand the component of strategic problem-solving tools which includes missions and shared visons, desired measurable results, obstacles, challenge statements and priority solutions. The training also assisted partners in developing a detailed one-year twinning partnership activity plan.

The piloting of the twinning partnership strategy has revealed the additive, synergy and antagonistic results of both partner districts. The results showed that partner districts shared tools and guidelines and were able to organize regular integrated supportive supervision and review meetings. Furthermore, through implementing components of collaboration functioning, both medium performing and low performing districts closed-in on achieving UHC, by addressing the root causes for all social inequalities in the availability, access, quality and burden of out of pocket payments.

The case study revealed that the twining partnership strategy, which was adopted from the WHO’s twinning partnership for improvement, helps the performance of the district health system to accelerate transformation towards UHC. This finding was consistent with Bitton et al.*,* (2017), Riggs et al.*,* (2014), Corbin et al.*,* (2012) and Argaw et al.*,* (2020) recommendations for strengthening primary health care as a pathway towards UHC [21, 28–30].

## Conclusions

Based on the results of the formative evaluation, the twinning partnership strategy piloted in eight districts of Ethiopia helped partner districts accelerate their performance towards fulfilling district transformation criteria and assisted them towards achieving UHC [9]. The twinning partnership strategy also helped district health systems to standardize services and build their leadership staff’s capacity which were fundamentally important in achieving results. Partner districts should be supported to be accountable and transparent to all stakeholders so as antagonistic results can be reduced. USAID Transform: Primary Health Care project, the Ministry of Health, Regional Health Bureaus and other development partners should support the scale up of this innovative performance improvement tool - twinning partnership - so that UHC can be achieved during the period of the Sustainable Development Goals.

## Supplementary information

**Additional file 1.** District (woreda) management standards self-assessment & validation checklist. The District management standards is a set of 26 management minimum standards with 81 verification criteria. The self-assessment and validation checklist is used to identify strengths and gaps, useful for performance improvements. The tool is used by both partner districts for performance management on a monthly basis. In addition, the zone health department organizes validation measurements on a semi-annual basis. The overall scoring is rated from 0.0 to 100.0%.

**Additional file 2.** Model village checklist. The overall scores on model villages will be converted out of 30.0%, which is assessed through model households, model schools improved latrine coverage, and skilled delivery coverage. The results will also contribute to district transformation criteria scores.

**Additional file 3.** Community-Based Health Insurance checklist. Community-Based Health Insurance household membership is calculated on an annual basis. Hence, the tool helps to organize memberships into new enrollments and renewals. Net active household membership scoring is rated from 0.0 to 100.0%. The results will be converted out of 30.0% and contributes to district transformation criteria.

**Additional file 4.** Key Performance Indicators checklist. The Key Performance Indicators are 18 in number. Each indicator is scored out of 100.0% but based on their importance, the Ministry set weights for each one. The overall score will be converted to 35.0% and will contribute scores to categorizing primary health care units.. The results will also contribute to high performing PHCU scores.

**Additional file 5.** Ethiopian Health Center Reform Implementation Guidelines (EHCRIGs) checklist. EHCRIGs are a set of 81 standards with 209 composite validation criteria. EHCRIGs assessment checklist is developed to perform self-assessment on a quarterly basis after which the district health office facilitates validation measurements on a semiannual basis. The performance management team and quality improvement committee use the information for performance and quality improvement initiatives. The overall scoring is rated out from 0.0 to 100.0% and translated into 35.0%.

**Additional file 6.** In-depth interview guide. The in-depth interview guide was developed after reviewing relevant literatures [17, 24]. The guide has three sections, an ice breaker, main question and probing questions.

## Abbreviations

BMCF: Bergen Model of Collaborative Function
CBHI: Community-Based Health Insurance
EHCRIGs: Ethiopian Health Center Reform Implementation Guidelines
FMOH: Federal Ministry of Health
MDG: Millennium Development Goal
MMR: Maternal Mortality Ratio
PHCU: Primary Health Care Unit
SDG: Sustainable Development Goal
SNNP: Southern Nations, Nationalities, and Peoples’
SPSS: Statistical Package for Social Science
UHC: Universal Health Coverage
USAID: United States Agency for International Development
WHO: World Health Organization
ZHD: Zonal Health Department
