# Supplementary material for: Accelerating the performance of district health systems towards achieving UHC via twinning partnerships
Source: BMC Health Serv Res. 2020 Sep 21;20:892. doi: 10.1186/s12913-020-05741-1 (PMC7507737; doi:10.1186/s12913-020-05741-1)
Supplement: Supplementary file 6 — Additional file 6. In-depth interview guide. The in-depth interview guide was developed after reviewing relevant literatures [17, 24]. The guide has three sections, an ice breaker, main question and probing questions. [file 12913_2020_5741_MOESM6_ESM.docx]

## **Additional file 6: In-depth interview guide (17, 24)**

**Introduction**

This tool was developed to collect data useful to accelerating woreda transformation: A formative evaluation of the twinning partnership strategy study in Ethiopia. The USIAD Transform: Primary Health Care project will analyze the data and synthesize the information for evidence-based decision making at various levels of the health tier system. The results of this study will be used to describe and explore the process of twinning partnership strategy implementation and related observed changes in the health system’s performance. Please note that the information you give us will be kept confidential and only used to fill in information gaps on the health system.

**In-depth interview guides**

Woreda health office staff and health center staff

**Icebreaker**

- - - 1. *How long have you been working in this organization?*
      2. *What are your roles and responsibilities?*

1. **Main question**

*“Would you please tell me the process you followed in implementing twinning partnership strategy at your organization?*

1. *Probing questions*

*Why do you think your organization joined in the twinning partnership strategy?*

*What is the mission or vision of the twinning partnership?*

*What kind of resources has your organization contributed to the partnership?*

*Who are the stakeholders engaged in the implementation? How do they contribute?*

*What financial resources are mobilized? From where?*

*What are the influencers (facilitators or barriers) in the implementation of the twinning partnership?*

*What kind of things support the twinning partnership\?*

*What hiders the twinning partnership?*

*Do you and your partners have clearly defined roles and responsibilities?*

*In what ways do partners communicate?*

*Who manages the twinning partnership?*

*Do you find the twinning partnership important? How?*

*In your experiences what are the achievements of the twinning partnership?*

*What motivates you to apply the twinning partnership?*

*What de-motivates you to apply the twinning partnership?*

1. *Only for twinning partnership training attendees*

*How did you find the introduction for twinning partnership strategy?*

*Have you observed changes in the health system as result of the twinning partnership? What are the changes?*

- *Production; maintenance; leadership; communication; structure/role and economy*

*How do you see twinning partnership before and after intervention?*

*Closing*

1. *Is there anything you want to tell me about twining partnership strategy? Can I share the transcript of this interview with you so you may add any additional opinions or experiences missed here? Do you have questions or suggestions for me (interviewer)?*
